# Assessment of Fecal Occult Blood Testing in Acute Hospital Settings: Perspectives of Internal Medicine Residents in a Multicenter Study

**DOI:** 10.7759/cureus.88524

**Published:** 2025-07-22

**Authors:** Suryanarayana Reddy Challa, Gabriel Buluku, Temitayo Gboluaje, Ahtshamullah Chaudhry, Angesom Kibreab, Farshad Aduli, Sneha Adidam, Hassan Ashktorab, Adeyinka Laiyemo

**Affiliations:** 1 Gastroenterology, New York University (NYU) Langone Hospital, Long Island, USA; 2 Gastroenterology, Howard University College of Medicine, Washington, DC, USA; 3 Internal Medicine, St. Dominic Hospital, Jackson, USA; 4 Gastroenterology and Hepatology, First State Gastroenterology, Dover, USA; 5 Internal Medicine, Howard University College of Medicine, Washington, DC, USA; 6 Medicine and Cancer Center, Howard University College of Medicine, Washington, DC, USA

**Keywords:** fecal occult blood test, inpatient, internal medicine, knowledge, postgraduate

## Abstract

Background

Fecal occult blood testing (FOBT) is an outpatient screening tool for colorectal cancer. However, it is widely used in the hospital setting among hospitalized patients for a myriad of reasons. The aim of our study is to understand the knowledge and current utilization of FOBT among internal medicine (IM) residents across multiple training programs in the United States.

Methods

This was a multicenter survey conducted among 25 IM residency programs. A 15-item questionnaire survey on FOBT was emailed to IM residents in May 2022, at the end of the academic year, with two bi-weekly follow-up reminders. Logistic regression models and Stata statistical software version 14.2 (StataCorp, College Station, TX) were used for the analysis. Those included in the study were IM residents at any level of training. The exclusion criterion was other residents not enrolled in an IM program.

Results

A total of 227 (100%) IM residents responded to the survey (n=96 (42.2%) in post-graduate year (PGY)-1, n=67 (29.5%) in PGY-2, n=64 (28.2%) in PGY-3). Overall, 151 (66.5%) residents sometimes or always ordered FOBT during their respective training period and 153 (67.4%) ordered this test more often in inpatient than in outpatient settings. Approximately 136 (60%) residents had knowledge of dietary restrictions but only 73 (32%) residents questioned the patients before ordering it. The triggers for ordering FOBT were mostly anemia (174, 32%) followed by a change in stool color (102, 19%), weight loss (99, 18%) and bleeding per rectum (71, 13%). Majority (141, 62%) of the respondents felt influenced by their supervisors and 130 (57.2%) felt that FOBT results will change their management. Overall, as postgraduate year training increased, trainees were less likely to order FOBT for suspected GI bleeding.

Conclusion

Our survey results showed that residents were influenced by their supervisors and ordered FOBT largely in the inpatient setting. Although there was noted improvement in understanding of the futility of FOBT in suspected acute GI bleeding, more than half of final-year trainees would still order FOBT. There is a need for better education of IM trainees in the utilization of FOBT.

## Introduction

Fecal occult blood tests (FOBTs) have been a fundamental component of colorectal cancer screening recommendations in outpatient settings for several decades. The available tests in clinical practice, guaiac-based fecal occult blood tests (gFOBT) and fecal immunohistochemical tests (FIT), collectively referred to as FOBTs, are designed to detect microscopic hemoglobin in stool but are prone to interference [1]. In the past decade, the Food and Drug Administration (FDA) has approved fecal DNA-based tests (FIT-DNA or Cologuard), which offer increased sensitivity and specificity for screening average-risk individuals [2]. Among these options, FOBTs are cost-effective and easily administered, making them widely utilized worldwide, often extending beyond their primary indication, especially in non-ambulatory settings.

Numerous studies have underscored the inappropriate use of FOBTs, failing to account for confounding factors such as diet, medications, non-gastrointestinal bleeding, and advanced age. This issue is particularly prevalent among physicians in the emergency department and on hospital floors, leading to higher rates of false-positive results. These inaccuracies can result in delayed care, impact clinical decision-making, lead to unnecessary gastrointestinal consultations, procedures, and added costs [3]. Physicians in hospital settings often request FOBTs when patients present with anemia and black stools [4,5]. According to a survey of physicians, a significant proportion of emergency medicine (EM) and family medicine (FM) physicians order FOBTs, with EM physicians often favoring point-of-care testing over relying on laboratory-based reporting [5]. Notably, only a limited number of studies have assessed the understanding of FOBT usage among internal medicine (IM) residents, revealing a lack of comprehension and certain misconceptions regarding its utility [6].

The null hypothesis of our study assumed that there is no association between the level of training of IM residents and their knowledge about the use and interpretation of FOBT. To this end, our study's primary aim was to evaluate the knowledge of IM residents at various training levels in selected residency programs across the United States, with a focus on their ability to appropriately use FOBT and interpret its results. In our assessment, the findings of this study can empower training programs to tailor their teaching methods, addressing the identified knowledge gaps. By doing so, these programs can ensure that their graduates are equipped with evidence-based practices before entering independent practice.

## Materials and methods

This multicenter survey was conducted among various IM residency programs across several states: Washington, DC, Pennsylvania, New York, New Jersey, Florida, Indiana, Illinois, Georgia, and California. The study targeted IM residents in postgraduate years (PGY) one, two and three, reflecting their graded levels of responsibility for patient care. The survey was initiated in May 2022, with bi-weekly follow-up reminders sent until the study concluded in June 2022. We considered the end of the academic year to be an ideal time to assess the knowledge and skills residents had gained throughout their training. The study was conducted in accordance with ethical guidelines and received an exemption from an institutional review board (IRB) review (IRB-2023-1191).

A 15-question survey instrument was developed by the authors and validated for its suitability to answer the research question by having it reviewed by four GI attending physicians and six fellows in the Division of Gastroenterology at Howard University, Washington, DC (see Appendices). The information gathered consisted of the level of training, frequency, and method of performing FOBT. The tool also assessed their knowledge of indications for the test, foods and drugs that may affect the test, and their confidence in interpreting the test results. Furthermore, respondents were evaluated for their clinical decision-making for a patient with a suspected GI bleed - whether they would first consult GI or order an FOBT.

The survey was distributed via email to IM residents using Microsoft Forms (Microsoft, Redmond, NY). The initial email, sent in May 2022, included a survey link, an invitation to participate, and a consent form detailing the study's purpose. Respondents were informed that their individual responses would remain confidential and not be visible to others. This approach aimed to ensure that participants provided answers based on their own knowledge and experiences, free from external influence. The collected data were extracted into an Excel (Microsoft, Redmond, NY) file and subsequently coded. We utilized logistic regression models and performed the analysis using Stata statistical software version 14.2 (StataCorp LLC, College Station, TX). A two-sided p-value of less than 0.05 was considered statistically significant.

*Inclusion criteria*: Internal medicine residents at any level of training.

*Exclusion criteria*: Any other resident not currently enrolled in an internal medicine training program.

## Results

Level of training and frequency of performing FOBT in acute hospital settings

A total of 227 responses were collected from 25 IM residency programs across the United States, comprising PGY-1 trainees (n=96, 42%), PGY-2 (n=67, 30%), and PGY-3 (n=64, 28%). In general, 151 (66.5%) residents reported that they sometimes or always ordered FOBT during their training, and 153 (67.4%) indicated that they ordered this test more frequently in inpatient settings compared to outpatient settings. The way residents performed this test varied, with 70 (31%) preferring digital rectal examination (DRE), 70 (31%) requesting stool samples, and 86 (38%) sending samples to the lab for FIT analysis. Notably, the likelihood of ordering an inpatient FOBT did not appear to significantly change as residents progressed through different levels of training (Pearson chi-squared test, χ²=1.2522, p=0.535) (Table 1).

**Table 1:** Comparison of resident physician responses by postgraduate (PGY) level FOBT: Fecal occult blood testing.

Questionnaire	PGY 1 (96), n (%)	PGY 2, (67), n (%)	PGY 3 (64), n (%)	Total respondents, n (%)	Pearson chi-squared test (χ²)	P-Value
In which setting have you ordered FOBT test more often?						
Inpatient	61 (64%)	48 (72%)	44 (69%)	153 (67%)	1.2522	0.535
Outpatient	35 (36%)	19 (28%)	20 (31%)	74 (33%)		
Does a positive FOBT change your management in inpatient setting?						
Yes	60 (63%)	42 (63%)	28 (44%)	130 (57%)	6.6568	0.036
No	36 (37%)	25 (37%)	36 (56%)	97 (43%)		
Have you ever felt influenced by other team members (including supervisors) to do FOBT?						
Yes	55 (57%)	46 (69%)	40 (62%)	141 (62%)	2.1715	0.338
No	41 (43%)	21 (31%)	24 (38%)	86 (38%)		
How confident are you in interpretation of positive FOBT result in any clinical scenario?						
Completely confident	5 (5%)	2 (3%)	3 (5%)	10 (4%)	5.5141	0.701
Very confident	23 (24%)	17 (25%)	22 (34%)	62 (27%)		
Somewhat confident	46 (48%)	36 (54%)	31 (48%)	113 (50%)		
A little confident	19 (20%)	9 (13%)	7 (11%)	35 (16%)		
Not confident at all	3 (3%)	3 (5%)	1 (2%)	7 (3%)		

Guidance and confidence level on performing FOBT among IM residents

In the inpatient setting, 141 (62%) respondents mentioned that they felt influenced by their team members, including supervisors, to order an FOBT test, and this trend remained consistent as the level of training progressed (Pearson chi-squared test, χ²=2.1715, p=0.338). Only 10 (4.4%) respondents expressed complete confidence in interpreting FOBT results, and this level of confidence did not significantly improve with increasing levels of training (Pearson chi-squared test, χ² = 5.5141, p = 0.701) (Table 1).

Knowledge and utilization of inpatient FOBT as the level of training progressed

Although 136 (60%) respondents were aware that certain foods or drugs might affect FOBT results, only 73 (32%) asked their patients about such factors before the test. The most common reasons for ordering an inpatient occult blood test included anemia (174, 32%), changes in stool color (102, 19%), weight loss (99, 18%), and rectal bleeding (71, 13%). Following the test results, 130 (57.3%) residents initially believed that a positive FOBT result would impact their patient management, but this perception shifted significantly as they advanced in their training, with PGY-3's being less inclined to believe it would alter management (Pearson chi-squared test, χ²=6.6568, p=0.035) (Table 2). In case of a suspected gastrointestinal bleed in a hospitalized patient, 128 (56%) respondents preferred obtaining an occult blood test over consulting a gastroenterologist. Interestingly, as the level of training progressed, PGY-3 residents were less likely to order an FOBT for a suspected GI bleed compared to PGY-1 residents (odds ratio (OR)=0.40; 95% CI: 0.21-0.77; p=0.006). However, 27 (42%) third-year residents would still consider ordering FOBT first. No significant difference was observed among second-year residents and interns (OR=0.76; CI: 0.40-1.4; p=0.410) (Table 2). Regarding the cost, 201 (89%) residents reported being unaware of the cost of FOBT in the inpatient setting.

**Table 2:** Comparison of preference for FOBT vs. GI consult for a suspected GI bleed by year of training FOBT: Fecal occult blood testing.

	For a suspected GI Bleed, which of the following would you consider first				
Postgraduate Year (PGY)	GI Consult	FOBT	Odds Ratio	95% Confidence Interval	p-Value
PGY-1	34 (35.4%)	62 (64.6%)	Ref	Ref	Ref
PGY-2	28 (41.8%)	39 (58.2%)	0.76	0.40 - 1.44	0.410
PGY-3	37 (43.6%)	27 (56.4%)	0.40	0.21 - 0.77	0.006

## Discussion

The use of FOBT was originally designed for outpatient colorectal cancer screening, primarily targeting average-risk individuals for the detection of colorectal neoplasia. Despite their lower sensitivity compared to other colorectal cancer screening modalities, stool-based tests have proven to be essential for achieving effective screening, reducing colorectal cancer (CRC) incidence and mortality [7-9]. It is worth noting that gFOBT requires dietary and medication restrictions, whereas FIT offers a more convenient alternative without such limitations [10-12]. However, careful patient selection is imperative since FIT should not be performed on high-risk individuals, those of advanced age, individuals with active hemorrhoidal bleeding, or those with new-onset inflammatory bowel disease (IBD) [13]. Our study findings uncover a concerning trend where these tests, which are relatively cost-effective, are inappropriately employed in inpatient settings without proper validation.

The technique employed to conduct FOBT significantly influences the test results. In office-based settings and emergency departments, a common method for obtaining stool samples is through a DRE, known to be highly inaccurate and prone to false-positive results for both gFOBT and FIT [14,15]. The interpretation of point-of-care testing varies significantly depending on the observer and testing environment, as demonstrated in a study by Selinger et al., where 12% of providers could not accurately interpret FOBT results [16,17]. Therefore, both the American Cancer Society and the United States Multi-Society Task Force on Colorectal Cancer recommend against using DRE for screening [14]. It is concerning that roughly 70 (31%) study participants employed DRE to obtain stool samples, emphasizing the need to address this practice to ensure more accurate and reliable outcomes.

Several retrospective studies, physician and residents' surveys conducted in the United States, Canada, and Australia have examined the utility of FOBT in acute hospital settings [4-6,18,19]. The frequency of FOBT orders in the hospital exhibited variation among specialties, with a higher frequency of tests being ordered by medical specialists, particularly within the FM department, as compared to surgical disciplines [5,20]. Common indications for performing FOBT in the hospital setting included evaluating iron deficiency with or without anemia, suspected gastrointestinal bleeding, the presence of dark stools, and suspicion of malignancy. However, these practices have shown no beneficial impact on clinical management. Instead, they have been associated with improper clinical decision-making, delays in care, unnecessary endoscopic procedures, and increased costs [4,18,19].

In a study assessing the use of FOBT for anemia in hospital settings, it was observed that there was no significant difference in endoscopic findings between patients with positive and negative FOBT results [20]. Moreover, evidence suggests that the sensitivity of FOBT in predicting the presumptive causes of iron-deficiency anemia (IDA) during endoscopy is as low as 58%, resulting in a significant number of false-negative tests for patients with identifiable causes of IDA [21]. Hence, the British Society of Gastroenterology's latest guidelines assert that FOBT offers no benefit in the investigation of IDA due to its insensitivity and lack of specificity [22]. Despite this guidance, our study revealed that 92.5% of respondents continued to order FOBT for the evaluation of anemia, reflecting a clear need for educational interventions to align practices with evidence-based guidelines.

Understanding the costs associated with ordering tests, especially off-label FOBTs, in the acute hospital setting is of paramount importance. An estimated average annual expenditure of $40,000, along with significant indirect costs incurred due to downstream management following positive FOBT results, was noted in a study by Gupta et al. [1]. Our survey revealed that a striking 201 (89%) study participants were unaware of the cost implications related to these tests. The reasons behind ordering these tests by residents in training are multifaceted, including practice habits, lack of knowledge of the costs involved, influence by their seniors and supervisors, lack of experience and fear of litigation from missed diagnosis [23]. These findings align with the observations from our study, highlighting a need for mentorship programs tailored to enhance trainee education.

Our study had some inherent strengths and limitations. One of its strengths lay in the inclusion of many IM resident participants at various levels of training, encompassing both university and community hospitals and conducting this study at the end of the academic year. Additionally, participants were ensured confidentiality, anonymity and were unaware of who else was participating in the study, thereby enhancing the reliability and validity of the data collected. However, the study was limited to IM residents in training, potentially missing out on important perspectives from the other specialties. Thus, the analysis and conclusions of our study findings should be interpreted in the context of such limitations.

## Conclusions

Our study unveiled a widespread practice of off-label FOBT use in acute hospital settings despite substantial evidence from the literature indicating its lack of value in this context. Our study reinforces the utility of educational interventions in addressing this misuse of FOBT testing, although other factors beyond the scope of this study may yet affect a provider's decision to order the test. Quality improvement initiatives aimed at restricting inpatient FOBT usage may provide an opportunity to find out interventions that are effective in the local context. These steps are crucial for shaping a more effective and evidence-based approach in the future.

## Appendices

**Table 3:** Survey Questionnaire

	Questionnaire:
1	What Postgraduate year do you belong to? o PGY-I o PGY-II o PGY-III
2	Choose your training program: o University/Hospital name
3	How often do you order an FOBT test? o Always o Sometimes o Rarely o Never
4	How do you perform the FOBT test? o Perform a DRE and do FOBT (guaiac smear) o Request a stool sample and do FOBT (guaiac smear) o Request a stool sample and send to lab for Occult blood test (ColoScreen or FIT)
5	Are you aware of any foods that might affect FOBT results? o Yes o No
6	Are you aware of any drugs that might affect FOBT results? o Yes o No
7	Have you ever asked patients about those specific foods that might affect the FOBT result prior to ordering the test? o Yes o No
8	Have you ever asked patients about those drugs that might affect the FOBT result prior to ordering the test? o Yes o No
9	Which of the following symptoms do you think warrants an inpatient FOBT test? (You may choose multiple) o Change in stool color o Bleeding per rectum o Abdominal pain o Weight loss o Diarrhea o Anemia
10	In which setting have you ordered the FOBT test more often? o Inpatient o Outpatient
11	For a suspected GI bleed in the hospital, which of the following would you consider first? o FOBT o Consult GI
12	Have you ever felt influenced by other team members (including supervisors) to do an FOBT in the absence of guideline-based indications? o Yes o No
13	Does a positive FOBT change your management in the inpatient setting? o Yes o No
14	Are you aware of the cost of the FOBT test? o Yes o No
15	How confident are you in interpretation of positive FOBT results in any clinical scenario? o Completely confident o Very confident o Somewhat confident o A little confident o Not confident at all
